# Supplementary material for: PPP1R35 is a novel centrosomal protein that regulates centriole length in concert with the microcephaly protein RTTN
Source: eLife. 2018 Aug 31;7:e37846. doi: 10.7554/eLife.37846 (PMC6141234; doi:10.7554/eLife.37846)
Supplement: Supplementary file 2. — Unless otherwise noted, all primers were used as a part of a Gibson Assembly based cloning strategy. [file elife-37846-supp2.docx]

| **Primer Description** | **Sequence (5’-3’)** | **Primer Name** |
| --- | --- | --- |
| Reverse primer for generating GFP-PPP1R35 in the pcDNA5 CMV vector (anneals vector) | CTTGTACAGCTCGTCCATG | ASGFPc7backr |
| Forward primer for generating GFP-PPP1R35 in the pcDNA5 CMV vector (anneals vector) | TCCGGACTCAGATCTGAATTG | ASGFPc7backf |
| Forward primer for generating GFP-PPP1R35 in the pcDNA5 CMV vector (anneals insert) | ggacgagctgtacaagATGATGATGGGTTGTGGG | ASGFPc7c7orff |
| Reverse primer for generating GFP-PPP1R35 in the pcDNA5 CMV vector (anneals insert) | cagatctgagtccggaCTACGCTTCCCATCGCCT | ASGFPc7c7orfr |
| Reverse primer for generating mutant GFP-PPP1R35 constructs in the pcDNA5 Sept2p vector from synthesized PPP1R35 mutant DNA strands (anneals vector) | GAATTCGGATCCGATATCAGC | AS-60 |
| Forward primer for generating mutant GFP-PPP1R35 constructs in the pcDNA5 Sept2p vector from synthesized PPP1R35 mutant DNA strands (anneals vector) | CTCGAGGGATCCACCGGA | AS-61 |
| Forward primer for generating mutant GFP-PPP1R35 constructs in the pcDNA5 Sept2p vector from synthesized PPP1R35 mutant DNA strands (anneals insert) | atatcggatccgaattcATGATGATGGGTTGTGGG | AS-62 |
| Reverse primer for generating mutant GFP-PPP1R35 constructs in the pcDNA5 Sept2p vector from synthesized PPP1R35 mutant DNA strands (anneals insert) | ggtggatccctcgagCTACGCTTCCCATCGCCT | AS-63 |
| Reverse primer for swapping the CMV promoter for the Sept2p promoter (anneals vector) | GGTGCCAAAACAAACTCCCATTG | AS-119 |
| Forward primer for swapping the CMV promoter for the Sept2p promoter (anneals vector) | ACCGGTCGCCACCATGGT | AS-120 |
| Forward primer for swapping the CMV promoter for the Sept2p promoter (anneals insert) | gagtttgttttggcaccCCTCCCGCGACCGCCTCT | AS-121 |
| Reverse primer for swapping the CMV promoter for the Sept2p promoter (anneals insert) | atggtggcgaccggtCCCGCCAAACAGCCCGAG | AS-122 |
| S45A PPP1R35 Site-directed Mutagenesis (forward primer) | cggcctggacttggccctgagcccgcgg | AS-149 |
| S45A PPP1R35 Site-directed Mutagenesis (reverse primer) | ccgcgggctcagggccaagtccaggccg | AS-150 |
| S47A PPP1R35 Site-directed Mutagenesis (forward primer) | ggacttgagcctggccccgcggcccgac | AS-151 |
| S47A PPP1R35 Site-directed Mutagenesis (reverse primer) | gtcgggccgcggggccaggctcaagtcc | AS-152 |
| S52A PPP1R35 Site-directed Mutagenesis (forward primer) | gcggctgaggggcgtcgggccgcg | AS-153 |
| S52A PPP1R35 Site-directed Mutagenesis (reverse primer) | cgcggcccgacgcccctcagccgc | AS-154 |
| Reverse primer for generating FLAG-BirA*-PPP1R35 (N-terminal tag) in the pcDNA5 BioID vector (anneals vector) | AGGCGCGCCGCCGCCGGG | AS-205 |
| Forward primer for generating FLAG-BirA*-PPP1R35 (N-terminal tag) in the pcDNA5 BioID vector (anneals vector) | GTACAGATATCTGCGGCCGCCTAACTCGAGTCTAGAGGGC | AS-206 |
| Forward primer for generating FLAG-BirA*-PPP1R35 (N-terminal tag) in the pcDNA5 BioID vector (anneals insert) | cccggcggcggcgcgcctATGATGATGGGTTGTGGG | AS-207 |
| Reverse primer for generating FLAG-BirA*-PPP1R35 (N-terminal tag) in the pcDNA5 BioID vector (anneals insert) | ccgcagatatctgtacCTACGCTTCCCATCGCCT | AS-208 |
| Reverse primer for generating PPP1R35-BirA*-FLAG (C-terminal tag) in the pcDNA5 BioID vector (anneals vector) | CCTATTGTACATATGGATCCGAG | AS-209 |
| Forward primer for generating PPP1R35-BirA*-FLAG (C-terminal tag) in the pcDNA5 BioID vector (anneals vector) | CGCGCCATAGCGGCCGCA | AS-210 |
| Forward primer for generating PPP1R35-BirA*-FLAG (C-terminal tag) in the pcDNA5 BioID vector (anneals insert) | atccatatgtacaataggATGATGATGGGTTGTGGGG | AS-211 |
| Reverse primer for generating PPP1R35-BirA*-FLAG (C-terminal tag) in the pcDNA5 BioID vector (anneals insert) | ggccgctatggcgcgCGCTTCCCATCGCCTCAG | AS-212 |
| Reverse primer for generating mCherry-RTTN in the pcDNA5 vector (anneals vector) | AGATCTGAGTCCGGACTTG | AS-265 |
| Forward primer for generating mCherry-RTTN in the pcDNA5 vector (anneals vector) | TGAGGCGCGCCATTAATTAAG | AS-266 |
| Forward primer for generating mCherry-RTTN in the pcDNA5 vector (anneals insert) | gctgtacaagtccggactcagatctATGGTCCTGGCAGGGCTC | AS-267 |
| Reverse primer for generating mCherry-RTTN in the pcDNA5 vector (anneals insert) | gatccttaattaatggcgcgcctcaGGAAGAATTAAGGAGCTGCACG | AS-268 |
| RNA Polymerase II qPCR Primer- forward | CCTTCGCTTACTGTCTTCCTG | AS-100 |
| RNA Polymerase II qPCR Primer- reverse | CTCCAGACGGCACAGAATATC | AS-101 |
| TATA-box binding protein qPCR Primer- forward | GAGAGTTCTGGGATTGTACCG | AS-102 |
| TATA-box binding protein qPCR Primer- reverse | ATCCTCATGATTACCGCAGC | AS-103 |
| *c7orf47* qPCR primer 1- forward | AAAGTCGTTCCAGATCCGC | AS-104 |
| *c7orf47* qPCR primer 1- reverse | AGCCAATTTCTCCCTGAGC | AS-105 |
| *c7orf47* qPCR primer 2- forward | CTCAGGGAGAAATTGGCTCTC | AS-106 |
| *c7orf47* qPCR primer 2- reverse | GGTGTGGAGATTCATAAAATAGCG | AS-107 |
