# Supplementary material for: PPP1R35 is a novel centrosomal protein that regulates centriole length in concert with the microcephaly protein RTTN
Source: eLife. 2018 Aug 31;7:e37846. doi: 10.7554/eLife.37846 (PMC6141234; doi:10.7554/eLife.37846)
Supplement: Supplementary file 3. — All siRNAs were from Ambion (by Life Technologies) except for RTTN that was from Thermo/Invitrogen. Upper case letters represent bases that are present in the target’s mRNA sequence. [file elife-37846-supp3.docx]

| **Target** | **Sense Strand Sequence (5’ to 3’)** | **siRNA ID** |
| --- | --- | --- |
| **Scrambled siRNA** | Sequence not available | 4390844 |
| **GAPDH** | Sequence not available | 4390850 |
| **PPP1R35, Exon** | CUGAGAAAGUCGUUCCAGAtt | s48124 |
| **PPP1R35, 3’ UTR** | GUAUUUUUGUGUUAAACUAtt | s195859 |
| **RTTN** | CCCGUGUCUUCACUUUGCAAUGGAA | RTTNHSS119506 |
